# Detecting Malicious Anomalies in Heavy-Duty Vehicular Networks Using Long Short-Term Memory Models

**DOI:** 10.3390/s25144430

**Published:** 2025-07-16

**Authors:** Mark J. Potvin, Sylvain P. Leblanc

**Affiliations:** 1 Department of Mathematics and Computer Science, Royal Military College of Canada, Kingston, ON K7K 7B4, Canada; 2 Department of Electrical and Computer Engineering, Royal Military College of Canada, Kingston, ON K7K 7B4, Canada; sylvain.leblanc@rmc.ca

**Keywords:** controller area network (CAN), J1939, anomaly detection, long short-term memory, deep learning, automotive security

## Abstract

Utilizing deep learning models to detect malicious anomalies within the traffic of application layer J1939 protocol networks, found on heavy-duty commercial vehicles, is becoming a critical area of research in platform protection. At the physical layer, the controller area network (CAN) bus is the backbone network for most vehicles. The CAN bus is highly efficient and dependable, which makes it a suitable networking solution for automobiles where reaction time and speed are of the essence due to safety considerations. Much recent research has been conducted on securing the CAN bus explicitly; however, the importance of protecting the J1939 protocol is becoming apparent. Our research utilizes long short-term memory models to predict the next binary data sequence of a J1939 packet. Our primary objective is to compare the performance of our J1939 detection system trained on data sub-fields against a published CAN system trained on the full data payload. We conducted a series of experiments to evaluate both detection systems by utilizing a simulated attack representation to generate anomalies. We show that both detection systems outperform one another on a case-by-case basis and determine that there is a clear requirement for a multifaceted security approach for vehicular networks.

## 1. Introduction

With exponential enhancements in artificial intelligence (AI) and developments in the Internet of Things (IoT), the world has become increasingly more connected, thus creating more nodes and generating more data [1]. Vehicular networks are made of such well-connected nodes. Both electric vehicles (EVs) and internal-combustion engine vehicles utilize a well-known communication protocol called the controller area network (CAN) bus. In 1986, Bosch designed and developed the CAN bus to be efficient, robust, and reliable to “support distributed real-time control with a very high level of security” [2]. While the safety of the CAN bus has been well demonstrated, this original claim to security is disputed by us and numerous other researchers who discovered that adversaries can access the CAN bus network remotely or directly with a physical link to carry out various forms of exploitation. In Ref. [3], Valasek and Miller outline numerous vulnerabilities that are prone to cyber-attacks, such as replay and masquerade attacks, as well as denial-of-service (DoS) attacks. These threats are equally menacing to the J1939 protocol used by heavy vehicles, which are the backbone of entire supply chains, such as getting produce from farms to markets to homes. The Society of Automotive Engineers (SAE) J1939 protocol is a particular standard that operates at a higher layer in the protocol communication stack and utilizes the CAN bus standard [4]. Thus, we contend that a task-tailored security system for the J1939 protocol would be more effective than one intended for the more generic CAN bus.

Since CAN bus, as a whole, has very limited security, there has been significant research and development aiming to provide better security through cryptography of protocol messaging and intrusion detection systems (IDS) that utilize machine learning and statistical analysis [5]. Despite the growing innovations in CAN bus research, there has been less focus on the higher layer protocol J1939, found in most commercial, heavy-duty trucks. The earliest that found mention of J1939 security was a paper in 2017 by Dr. Butler discussing the concept of utilizing machine learning classifiers to detect packet-based injection attacks [6]. Later that year, Mukherjee et al. [7] became the first to detect message injection attacks on J1939 networks using an ingenious method based on graph theory. The notion of cryptography was in consideration for securing the J1939 protocol using various authentication and encryption designs [8,9], respectively; however, the sheer communication overhead required did not make these solutions feasible. In 2019, Shirazi et al. [10] proposed detecting anomalies in J1939 networks by experimenting with seven different machine learning classifiers using a “five-fold cross-validation approach to avoid overfitting”. Our work is similar to that of Shirazi et al. in that both approaches utilize machine learning concepts; however, we use design features that are significantly distinct from those described in [10], as we will describe in detail in the upcoming sections. For instance, Shirazi et al. utilize the full 64-bit data payload to train their machine learning algorithms, like a CAN bus detection system, whereas our approach breaks down the payload into its respective sub-fields to train Long Short-Term Memory (LSTM) neural network models. Moreover, the attack scenario used in Ref. [10] imitates a compromised ECU node that injects malicious packets onto the bus as a means of causing aperiodic frequency effects, whereas the attack representation we use simulates modifying the Data field of the packet directly without causing any frequency-based anomalies. Several years later, Jichici et al. pushed the innovative yardstick further by designing a multi-layered application specifically for SAE J1939 commercial vehicles [11] by using a one-way encryption function to ensure authentication and a lightweight cipher on the Data fields as a means for intrusion detection. Most recently, in early 2025, a paper was published that describes a “next generation stateful firewall” specifically designed for the J1939 network, which can detect and prevent threats based on rules and can block or allow traffic based on context using threat intelligence [12].

The aim of our research is to utilize deep learning, specifically LSTM neural network models, to design a tailor-made J1939 IDS and compare it against the CAN bus IDS employed by Taylor et al. in Ref. [13], which also trained LSTM models. In order to ensure a controlled comparison, we utilized the attack framework from Ref. [14] and generated synthesized attack data from eight hours of driving time from a 2014 International ProStar Class 8 truck over four days in various driving conditions. The data were segregated accordingly to train and test the LSTM models to compare the J1939 and CAN detection systems. In Ref. [13], Taylor et al. designed a CAN detector and evaluated it across multiple model types, such as deep learning and Markov chain models. We focused solely on LSTM units as this design was the most effective method for data sequence anomaly detectors (ADs) in their work. Moreover, our research seeks to improve upon J1939 security by determining the optimal approach of a more application-specific defense system for heavy-duty vehicles through the comparative analysis of physical layer CAN detectors and higher layer J1939 detectors.

The remaining sections of this paper are as follows: Section 2 discusses some of the key background information on the CAN bus network, the J1939 protocol, deep learning concepts, as well as the performance metrics used to assess the ADs. Section 3 describes details of the data analysis we used in pre-processing and feature selection, the hyper-parameter optimization process, an explanation of the architecture of the LSTM model, and an overview of the attack model used. Section 4 outlines the comparative results of both detectors using various performance metrics. Section 5 provides future work recommendations and final summations.

## 2. Background

The CAN protocol is a widely used network communications protocol that is reliable and efficient. It is employed by the automotive and trucking industry, and it is the network protocol that controls communication packets between Electronic Control Units (ECUs) in vehicles. “[It] is a protocol for multi-master, serial, high-speed communication along a bus [and] it is designed for safety-critical and real-time applications” [15]. All ECUs within a vehicle communicate with each other through the CAN bus internally; with some ECUs communicating externally to other outside interfaces, such as 3G/4G, Bluetooth, and GPS [16]. These outward-facing entities are external attack vectors that can be exploited by a sophisticated adversary. An internal avenue of infiltrating the CAN bus is by physically connecting to the network via the On-board Diagnostic (OBD) port or Universal Serial Bus (USB) ports, the latter being found in more modern vehicles.

### 2.1. CAN Message Format

**Figure 1:**
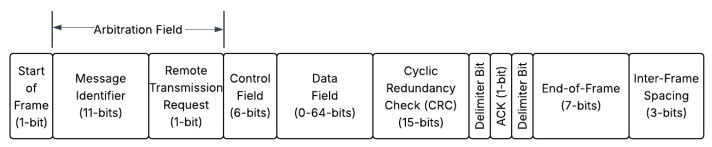
CAN bus message frame.

The CAN is a broadcasting protocol where ECUs publish a message in the form of a packet received by all other ECU nodes connected to the same CAN bus. The nodes filter the packet to determine if they are the intended recipients; if they are, they strip the packet header to uncover its payload; otherwise, the packet is ignored. CAN messages have a maximum transmission rate of 1 Mbit/s, which translates to a maximum of approximately 7000 packets sent over the bus per second [17]. Figure 1 illustrates a standard CAN (CAN 2.0A) message frame. The CAN message consists of a series of bits that correspond to specific fields, where dominant bits are 0 s and recessive bits are 1 s [18]. The key fields include the Message Identifier (ID), Control (Data Length), Data, and Cyclic Redundancy Check (CRC). The Message ID is contained within the Arbitration field, and this denotes the destination (recipient) as well as the priority of the message. For instance, a message intended for a node with a lower ID would gain arbitration and have priority over any messages sent to a node with a higher ID. The Control field contains a bit for the Identifier Extension (IDE), which is set when the message is extended, a reserve bit, and 4 bits for the data length of the payload, which is up to 8 bytes in size. The Data field contains the actual payload intended for the recipient node. Moreover, the CRC contains the checksum for the entire packet and confirms the integrity of the message.

Other key fields in the message include the Remote Transmission Request (RTR) bit, which is set to one when the sender requires information from another node. There is also the Start of Frame (SOF) bit that “marks the start of a message and is used to synchronize the nodes on a bus after being idle” [18]. The End of Frame (EOF) is a 7-bit recessive field that signifies the end of the CAN message.

**Figure 2:**
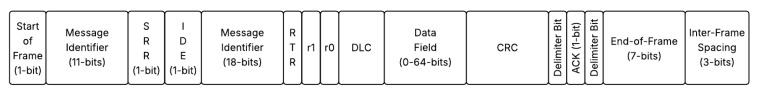
Extended CAN bus message frame.

The Extended CAN (CAN 2.0B) message is like a standard message with a few additional fields, as shown in Figure 2. It contains an additional ID field of 18 bits, which is preceded by the Substitute Remote Request (SRR) bit that is set to 1 and substitutes the RTR bit. This is followed by a recessive IDE bit that signifies an extended message. Lastly, an additional reserve bit (r1) is added before the Data Length Code (DLC). The purpose of an extended message is to allow for flexibility and scalability as the larger number of ECUs in vehicles increases the overall complexity.

### 2.2. SAE J1939 Message Format

The SAE J1939 protocol utilizes only the CAN 2.0B structure with a 29-bit ID, and it is capable of speeds up to 250 Kbit/s [19]. At this speed, a typical J1939 bus generates approximately 1000 packets per second. When a CAN message is sent over the bus and becomes unpackaged at the J1939 application layer, the recipient only views the timestamp, ID, and data of the message. These are the three key parameters utilized throughout our research.

The J1939 message format follows the exact same structure described in the previous section with the 29-bit extended ID. The J1939 ID is broken down into three sub-fields: Priority (3 bits), Parameter Group Number (PGN) (18 bits), and Source Address (8 bits). The Priority sub-field corresponds to the arbitration process applied to every CAN message to determine its priority on the bus. Thus, a Priority of 0 (0b000) is the highest priority, whereas a Priority of 7 (0b111) has the lowest priority. The PGN is the next sub-field of the ID, and it can provide more granularity as to the functions of each ECU, such as Engine Temperature 1. The last eight bits of the ID are the Source Address, which is the address of the sender node. All nodes on the bus have a distinct address, which is independent of PGNs, meaning that each ECU node on an active bus contains a PGN specific to its functionality as well as a source address for network management.

The last vital field of the J1939 message is the Data field; this 64-bit field contains the Suspect Parameter Numbers (SPN) sub-fields, which encompass the key information that the J1939 packet is transmitting, such as Engine Coolant Temperature, Fuel Temperature, etc.

Thus, each PGN (found in the extended ID) corresponds to several SPN sub-fields (found within the Data field). The SAE J1939 Standard [4] contains a master list of approximately 200 PGNs and 1000 SPNs that are set as standard across all J1939 protocol-equipped heavy-duty trucks. However, there are J1939 original equipment manufacturer (OEM) products that vary depending on the truck make and model; these contain PGNs and SPNs that are proprietary and not part of the standard. Despite this fact, there is a substantial amount of information in Ref. [4] that an adversary can easily access and which potentially allows them to gain information, allowing them to craft malicious packets and insert them onto the bus to detrimental effects. Hence, for the purposes of our work, we only use the PGN field from the ID and the corresponding SPN sub-fields from the Data field.

### 2.3. Deep Learning Approach to Detecting Anomalies

With an understanding of the CAN bus network and its data, we can look more closely at the motivation behind using deep learning models for our J1939 detection system. In recent years, deep learning models have been used to detect anomalies in large data sets, specifically data sequences. Malhotra et al. used LSTM networks to accurately detect anomalies in time series data [20]. Moreover, deep learning models have continued to be a proven technique for anomaly detection in ordered sequences of discrete data for high-dimensional problem sets [8,20]. As we discussed above, these approaches are starting to gain traction in heavy-duty vehicle networks, such as the ground-breaking work by Shirazi et al. [10]. In addition to the factors related to LSTM modeling and anomaly detection, coupled with the success of Taylor et al.’s research in Ref. [13], we assert that our J1939 detection system with LSTM neural network units trained on individual SPN values (data sub-fields) will outperform a generic CAN bus detection system using LSTM units only trained on PGN values (full 64-bit Data fields).

Because of the limited size of our dataset, we had to be very systematic to optimize the number of test cases of simulated attack data used to evaluate our models. The attack framework used for our experiments, which will be explained in more detail in Section 3, is based on the design used by Taylor et al. in Ref. [14], which implements a replay attack and a data modification attack.

### 2.4. Performance Evaluation—Measure of Effectiveness

As we repurposed the CAN detector from Ref. [13] for J1939 networks and compared it to our proof of concept J1939 detector, there was a requirement for a clear and concise form of evaluation to accurately compare both systems. A generally accepted definition of effectiveness is that it is the quality that ensures the system can complete the task it is intended to accomplish. An anomaly detection system is evaluated based on its ability to correctly label data as either benign or anomalous. In our context, we define effectiveness as the ability of a system to achieve the desired results, which pertains to yielding high detection rates (i.e., accurately identifying anomalies) while keeping low false positive rates, as defined below. Furthermore, each AD model outputs a score based on the input data sequences, and the scores indicate if the input was anomalous. Assuming the AD is effectively trained, the scores between normal and anomalous inputs will be easily differentiated so that the system can decide whether to label the data as benign or anomalous. With this labelled data, we can calculate various performance metrics to determine the system’s level of effectiveness. Pandey suggests that the area under the curve (AUC) value is a feasible, singular output score to determine the effectiveness of an AD model [21]. We will describe the various performance metrics and calculations required to calculate the AUC value throughout this sub-section.

Since we are comparing two different detection systems, it is important that we minimize bias and maintain the integrity of all control variables. Each system evaluated is programmed to generate an error score based on the comparison of the predicted bit to the actual bit next in the data sequence. The scoring system is strictly based on each individual model, optimized to achieve the best results in isolation. For our setup, we decided to mirror the environment created by Taylor et al. in Ref. [13] to effectively compare results.

The final singular score of each model is specifically based on the following settings: error type and output score type. The error type is the loss function of the LSTM model; for our purposes, log loss was used for both detection models, as research indicated that it was preferred for hyper-parameter optimization and overfitting prevention [22,23]. The output score type is the process of converting the vector of scores outputted from the model into a single score for each specific input sequence. For this setting, it is important to account for “the noisiness of individual bit losses against the importance of a single bit’s score as an indicator of an anomaly” [13]. Thus, we utilized the same scoring methods used by Taylor et al. in Ref. [13]: maximum, average, rolling window mean, and log sum. The first two are simply the highest value and the calculated mean in the sequence, respectively. The rolling window mean is determined by calculating the average over 100ms intervals and taking the maximum score, whereas the log sum is determined by finding the log of each value and taking the average score.

Since our detection models predict the next data sequence in the network traffic to determine whether a packet is anomalous, it must be able to accurately label benign or anomalous traffic using the following classic designators:

- True positive (*t_p_*): detector identifies an anomaly correctly;
- False positive (*f_p_*): detector incorrectly labels benign traffic as anomalous;
- True negative (*t_n_*): detector identifies benign traffic correctly; and
- False negative (*f_n_*): detector incorrectly labels anomalous traffic as benign.

With these definitions, we can now compute key metrics to gain an understanding of the strength of each detection system. *Precision* is defined as true anomalies correctly identified proportionate to all anomalies labelled by the model [24], it can be expressed as

(1) $$\mathrm{Precision}=\frac{t_{p}}{t_{p}+f_{p}}$$

A *precision* of 1 indicates that the detector identified all anomalous data correctly and, thus, has a zero false positive rate, whereas 0 shows that no anomalies were identified accurately, which yields a zero true positive rate. Moreover, *recall* is defined as accurately predicted anomalies proportionate to all anomalies and expressed as [24]

(2) $$Recall=\frac{t_{p}}{t_{p}+f_{n}}$$

A *recall* of 1 indicates that all anomalies were correctly labelled, whereas 0 shows that no anomalies were identified. *Recall* is also referred to as sensitivity or true positive rate. *Accuracy* is described as the rate in which the detector is correct in its labeling of normal and anomalous traffic [24]. It is mathematical defined as

(3) $$Accuracy=\frac{t_{p}+t_{n}}{t_{p}+f_{p}+t_{n}+f_{n}}$$

An *accuracy* of 1 logically shows that the detector correctly identified all data whereas 0 shows that it was wrong in all labeling.

Along with these performance metrics, one must also consider the setting of thresholds; this relates to the rate of false alarms by the detection system deemed acceptable. Threshold settings are dependent on the severity of the experiment with safety or risk to life playing an important role. This is applicable to the J1939 network as there are ECUs that are vital to safety, such as the Airbag Control Unit and brakes, as well as less significant ECUs, such as power locks. Using *precision* and *recall*, we are able to calculate the F-score, which is defined as “the weighted harmonic mean of the test’s precision and recall” [25]. It is defined by the following calculation:

(4) $$F_{\beta }={\left(1+\beta \right)}^{2}\times \frac{precision\times recall}{\left(\beta^{2}\times precision\right)+recall}$$

We calculated *F_β_* scores, where *β* = 0.1, as this approach is widely used as to emphasize performance on precision rather than recall and, thus, we set beta to 0.1 based on the results in Section 4 [20].

As mentioned earlier, the LSTM model output scores are based on whether it identifies the input sequence as normal or anomalous. As such, a decision threshold must be set for each unique case based on the model ID, error type, and score type; this requires that we optimize our metrics for each test case of both detection models. Based on our research using the Scikit-Learn 0.22.2 Python 3.7 library [26], we set our threshold so that we maximize the F*_β_* score. The threshold setting is based on the acceptability level of false alarms. In essence, if our detection system identifies every sequence as anomalous, then it technically labels every anomalous data correctly; however, it also falsely labels every benign sequence as anomalous. Hence, the choice of the decision threshold is a decision that has a significant impact on the effectiveness of detection models. In both our J193 proof of concept detection system and the CAN bus detection system from Ref. [13], test cases were all conducted off-line and not in real-time when the vehicle is in motion. Even though our work is designed for off-line analysis, the results still provide a significant contribution regarding the design and implementation of an effective detection system for J1939 networks. Our experimental comparison of CAN and J1939 detectors using strictly J1939 network traffic allows us to recommend a priority of effort in protecting vehicular networks at lower layers (i.e., physical CAN) or higher layer protocols (i.e., J1939 application) or a combination of both. Therefore, it is important that we compute a standardized score for each detection system to evaluate and compare our models.

Once the data have been processed and calculations are completed, we next plot the Receiving Operating Characteristic (ROC) curve. The ROC curve and its associated AUC value incorporate true positive and false positive rates, and they “are important evaluation metrics for calculating the performance of any classification model” [21]. This statement holds true for anomaly-based detection models as well. The ROC curve is a scatter plot of true positive rates on the y-axis and false positive rates on the *x*-axis with a fluctuating threshold setting. The actual area under this curve, calculated through integration, yields the AUC value, which provides us with a scoring value that indicates the success of the model. A perfect anomaly detector has a 1.0 rate of true positives and a 0.0 rate of false positives, which yields an AUC value of 1.0, whereas the worst detector has a ROC curve of a straight line (i.e., *y* = *x*) and an AUC value of 0.5. Moreover, without loss of generality, we assert that an AUC of 0.75 is a mediocre detection system. Therefore, the anomaly detection system with an AUC value closest to 1.0 is deemed more effective in this experimental environment.

### 2.5. Performance Evaluation—Measure of Efficiency

As another means of evaluating performance, the efficiency of our detection models is secondary to effectiveness; however, it is still an important factor in our experiment. The training of LSTM models takes a significant amount of time and computing power, especially when it comes to the task of adjusting hyper-parameters and subsequently fine-tuning the models to ensure we are using the appropriate number of layers and neurons, batch size, dropout rate, as well as loss and activation functions, to achieve the optimal results. Even for an asynchronous detection system, it is crucial to be as efficient as possible when it comes to training deep learning models. All our LSTM models were trained successively on a Windows 10 workstation with an NVIDIA GeForce GTX TITAN GPU. We used the NVIDIA CUDA Toolkit to develop, optimize, and deploy our GPU-centric training environment in conjunction with using Keras 2.3.1 and TensorFlow 2.1.0 to build and train our LSTM models. Due to the numerous parameters involved in training LSTM models, we conducted an optimization task on one CAN detection model ID (for PGN 0×F002) and one J1939 detection model ID (for SPN 191) to determine the appropriate settings for each type of detector. The total training time for each optimization task was 182 h for the CAN model and 67 h for the J1939 model. Thus, we utilize the above performance metrics to conduct a comparative analysis between the J1939 and CAN bus anomaly detectors to definitively determine the more effective security system for heavy-duty vehicles.

## 3. Intrusion Detection System Architecture

In this section, we detail the data used for our experiments, how they are analyzed, preprocessed, and then fed into the LSTM models, and how they are trained through the process of hyper-parameter optimization and backpropagation. We also outline details of the attack model used to test both detection systems and provide a detailed outline of the architecture design.

Our approach to working with captured traffic was first to analyze the raw logs thoroughly and methodically. The J1939 traffic used in the experiments was captured from a Class 8 truck, courtesy of Defence Research and Development Canada (DRDC) in Valcartier, QC, Canada. Approximately eight hours of driving time were captured for the purposes of this research throughout the Valcartier training compound in a controlled traffic environment. The traffic captures equate to around 10 million packets, which ensured we had enough data to train our deep learning models as well as to enable the process of feature selection. The full traffic logs contain a total of 107 distinct IDs, each with a full 64-bit data payload. Of the 107 IDs, 68 are captured under the SAE J1939 Standard and the rest are likely proprietary. Burakova et al. [27] focused their attacks on engines and transmission, as these aspects of heavy-duty vehicles could result in detrimental effects if they were to be compromised. Thus, this research focuses on the PGNs that pertain to those control units (i.e., PGN 0×F002 Electronic Transmission Controller 1, 0×F003 Electronic Engine Controller 2, 0×F004 Electronic Engine Controller 1, and 0×F005 Electronic Transmission Controller 2).

### 3.1. J1939 Data Selection

**Figure 3:**
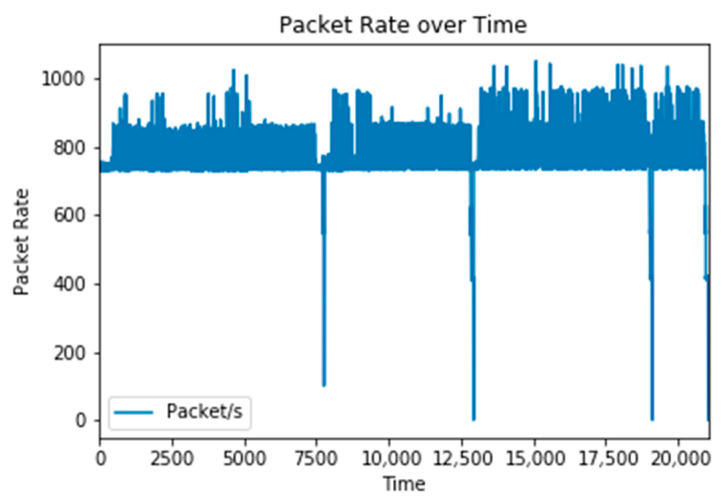
Packet rate of the entire dataset.

While we focus on the payload of J1939 packets to detect anomalies, there is still significant value to analyzing packet transmission rates. This analysis provides us with insight to ensure that the dataset is consistent enough to sufficiently train the CAN and J1939 detection models. Figure 3 illustrates the rate of packets per second for the full traffic logs. Based on the clustering of packet rates, we can conclude that the majority of the J1939 traffic generates between 700 to 850 packets per second.

Next, we must ensure that our selected features yield datasets of sufficient size and differentiation to efficiently train our LSTM models on the normal behavior of J1939 traffic. Table 1 displays the total number of packets as well as the Data field variability for our selected PGNs. The ‘# of Bits Changed’ column indicates the total number of specific bit positions from the 64-bit payload length that changed across the entire traffic capture. As the models were trained at the individual bit level for each PGN/ID, any bit position that did not change throughout all the traffic logs was discounted from the analysis. This technique was used for both the CAN and J1939 detectors.

**Figure 4:**
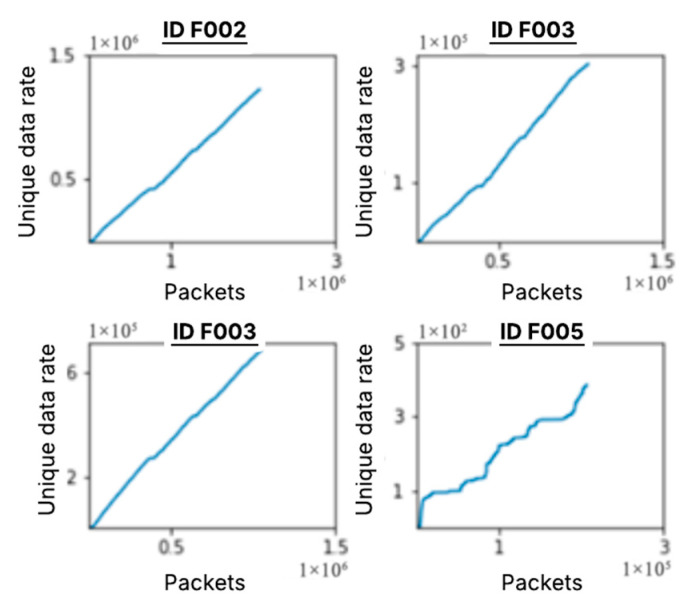
Unique data rate of growth for select PGNs.

To further illustrate the data growth, Figure 4 shows the unique data rate change over the course of the 8-h trip, and we can observe that the four PGNs selected for our experiment have a steady growth rate. As we see an upward trend in the rate of unique words for the other four PGNs, we forecast that this growth will continue for an extended period. It is also important to note that ID 0×F005 has a significantly lower unique %, as indicated above in Table 1, but it was included in our test case for both detectors because of its relation to the other IDs and its positive correlation in Figure 4.

Table 2 outlines the complete list of IDs (i.e., SPNs) used for our J1939 anomaly detection system mapped against their corresponding PGNs used in the CAN AD.

### 3.2. Long Short-Term Memory Neural Network Models

**Figure 5:**
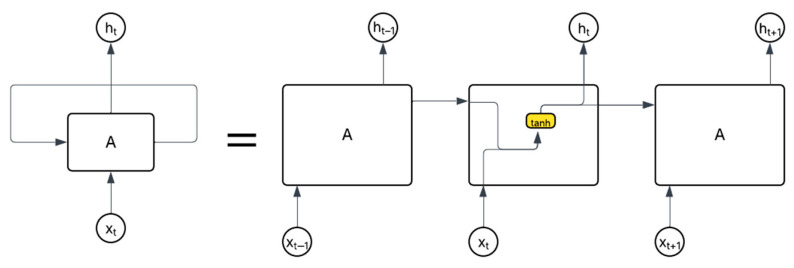
Unrolled recurrent neural network.

Deep learning models have been used to detect anomalies in large datasets, specifically data sequences. With our dataset analyzed and processed, it can now be fed into our specific neural models. Since LSTM units are a type of recurrent neural network (RNN), the input data utilize statuses from previous states as inputs and then transmits the corresponding output to the next layer of the model until the final layer is reached and the resultant output is given. Figure 5 displays this idea with *A* representing a neural network node with an input, *x_i_*, and output, *h_i_*, as well as a single *tanh* layer. The hyperbolic tangent, or *tanh*, is an activation function that scales arbitrary data within the range [−1, 1]. The chain-like structure in a RNN model makes it very effective for predicting the next values in data sequences [28]. RNNs follow a specific calculation to compute its output. Mathematically, each node of an RNN takes the following form:

(5) $$h_{t}=tanh\;(W\times x_{t}+U\times h_{t-1})$$

where *W* and *U* are weight matrices, *x* is the input, and *h* is the state/output of the cell, while *tanh* squeezes the output between −1 and 1 as described above. The current state of a node is dependent on the previous state and the input.

RNN models utilize several hidden layers, each containing multiple nodes, to make these predictions and apply error calculations to determine the accuracy of their estimations. The result of the error measurement is then used to re-train the model by adjusting the weight matrices in (5) above; this process is called backpropagation.

**Figure 6:**
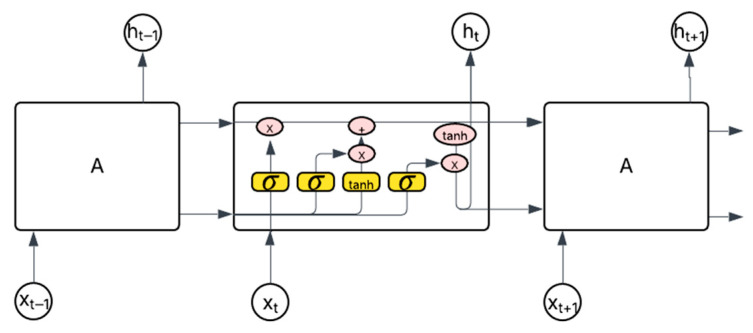
Unrolled LSTM unit.

The method of backpropagation updates the weight matrices at each layer going backward from the output layer to the input layer. This adjustment causes the error value to shrink or grow exponentially as the updates move back, causing the weight matrices to be inaccurately updated causing falsified predictions; this issue is referred to as the vanishing gradient problem as described by Hochreiter and Schmidhuber in Ref. [29]. LSTM units are used to “overcome [the] error back-flow problems” of RNNs and are capable of improving the accuracy of data sequence predictions, making them a suitable option for our application [20]. LSTM units utilize key features, such as input and forget gates that control the internal state of that layer, which also depends on the previous state and the new input. During training of these models, the weight matrices, *W_i_*, are updated through an adjustment based on the loss function and thus minimize error [29]. Conceptually, they have the same repeating modules that we saw associated with generic RNNs in Figure 5. However, they contain four collaborative internal layers that use gates to facilitate the model’s ability to remember and forget past states based on the input, *x_i_*, and previous cell’s state, *h_t_*. Mathematically, there are significant computations taking place within node A. In Figure 6, the yellow boxes represent neural network layers, from left to right; we observe the forget gate layer first, which can be expressed in Equation (6).

(6) $$f_{t}=\sigma \;(W_{f}\cdot \left[h_{t-1},x_{t}\right]+b_{f})$$

where *W_f_* is a weight matrix and *b_f_* is a bias term. The sigmoid function, *σ*, is an activation function and scales the output to [0, 1]. For this gate, if *f_t_* approaches 1, then it is more likely to be kept, whereas the closer it approaches to 0, it increases the likelihood it will be dropped.

Next, referencing the two middle yellow boxes in Figure 6, we observe another *σ* function paired with a *tanh* function, which represent the input layer, *i_t_*, and a vector of new candidate states,$\;\tilde{C}$_t_, respectively. These are expressed in

(7) $$i_{t}=\sigma \;(W_{i}\cdot \left[h_{t-1},x_{t}\right]+b_{i})$$

(8) $${\tilde{C}}_{t}=tanh\;(W_{c}\cdot \left[h_{t-1},x_{t}\right]+b_{c})$$

where *W_C_* and *W_i_* are weight matrices and *b_c_*, *b_i_* are bias terms. These calculations are used to determine the information that is stored in the new cell state, *C_t_*, which replaces the forgotten value as determined by f_t_.

(9) $$C_{t}=f_{f}\cdot \;C_{t-1}+i_{t}\cdot \;{\tilde{C}}_{t}$$

Lastly, there is a sigmoid layer that is used to determine the cell state that will be outputted. The output, *o_t_*, controlled by a weight matrix, *W_o_*, is updated through backpropagation and a bias term, *b_o_*.

(10) $$o_{t}=\sigma \;(W_{o}\cdot \left[h_{t-1},x_{t}\right]+b_{o})$$

(11) $$h_{t}=o_{t}\cdot tanh\;(C_{t})$$

### 3.3. LSTM Architecture and Hyper-Parameter Optimization

The neural design of LSTM models is multi-faceted, complex, and characterized by many parameters. Bengio states that the task of determining hyper-parameter settings for deep architectures is an extremely difficult and resource-intensive optimization problem space [30]. With a strict budget of time and computing power, random search is a proven effective and efficient method to identify appropriate parameter values since most parameter do not heavily influence the overall result [22]. These parameters are preconfigured when training LSTM models and one can then examine the results, as we have shown in the tables below. Moreover, the dropout rate, the portion of data randomly ignored during training, is another critical factor used in training neural networks as it aids in the prevention of overfitting [23]. Overfitting occurs when a model trains well on the training data but does not generalize strongly on the validation dataset [24]. For our experiments, the dropout rate was used to fine-tune the models to ensure that they were trained effectively. Table 3 outlines the results of the 10 iterations of the random selection approach we used to identify suitable hyper-parameters for our J1939 detector. Based on our analysis of the results, we determined that the optimal settings for our SPN model is a batch size of 16, three hidden layers of 64 neurons, an input sequence size of 10, and dropout rate of 0.2 and 0.5. This last parameter has two optimal values as the regularization of certain LSTM models was required to counteract overfitting. Since we only conducted our optimization task on SPN 191, when extrapolating the results for the other 11 SPNs, we found that the calculated loss for the training set and validation set did not always correspond properly. Thus, we investigated the irregularity on a case-by-case basis and determined that by experimenting with the dropout rate, we were able to counteract overfitting.

Table 4 displays the outcome of the 10 training cycles of our hyper-parameter optimization task for the CAN detector. From the results, we observe that the optimal settings consist of a batch size of 64, three hidden layers of 512 neurons, an input sequence of 40, and a dropout rate of 0.2 and 0.4. Like our SPN models, additional tweaking was required as the above settings caused some other PGN models to overfit and generalize the training dataset.

As we see in Table 3 and Table 4, there are no perfect solutions, with low training and validation losses (<0.10) or high accuracy ratings (>90%). This observance suggests to us that there is room for future work in optimizing LSTM models to achieve higher performances.

LSTM model performance can also be dependent on the shape of the input vector. The number of bits inputted into the models is drastically different between our J1939 detector and the CAN detector used by Taylor et al. [13]. For the former, since we are just focusing on specific SPN sub-fields, the input dimension is no more than 16 bits, whereas the latter concentrates on the full data payload of 64 bits. Since both models are configured at the bit-level, any bit position that does not change throughout the entire dataset is dropped from the experiment. Thus, the input size dimension of our J1939 models is up to 16 bits, and the CAN models are up to 64 bits.

Other settings may be fine-tuned by randomization; however, based on our binary dataset, research suggests specific settings for other parameters, such as loss function and optimizer. For instance, the loss function is a tool in modeling that essentially calculates how bad a model is based on its predictions compared to the actual value, aiming to minimize this difference. As mentioned above, refs. [22,23] indicate that the log loss function, or binary cross-entropy in Keras, is the most suitable loss function for neural networks. In training deep learning models, an optimizer is used to speed up the process; while there are several options, we implemented the Adam optimizer [31] for its simplicity and efficiency. Moreover, the activation function is a parameter setting in deep learning models that scales data to a particular scope. The rectified linear unit (ReLU) function is the recommended hyper-parameter for the hidden activation as it is fast and effective [24].

**Figure 7:**
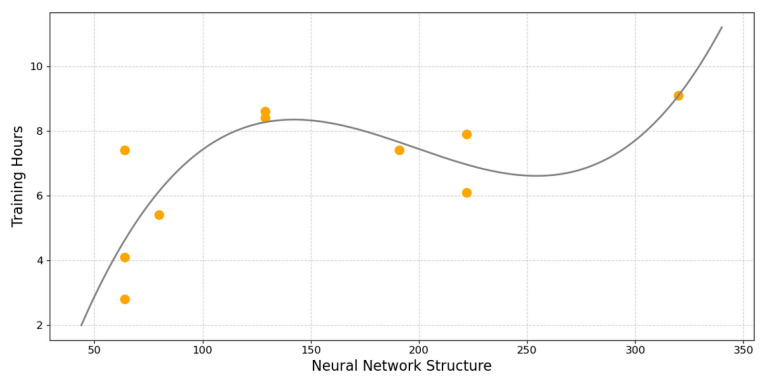
Relationship between network complexity and training time.

To ensure that training did not go on indefinitely, we factored in a stopping condition called patience [32] into the building of our models, where the training would cease if there were no improvements to the training loss after eight epochs in order to optimize our resources. Figure 7 illustrates the relation between the complexity of an LSTM network and its effect on training time with a 4th-degree polynomial fit curve using data from 10 iterations of hyper-parameter optimization on SPN models of the J1939 AD. The x-axis, Neural Network Structure, is defined by the sum of the total number of neurons as a product of the number of layers and the number of neurons per layer. The positive correlation of this graphic provides us with a clear depiction that the greater the depth of a network, the longer it takes to train it.

**Figure 8:**
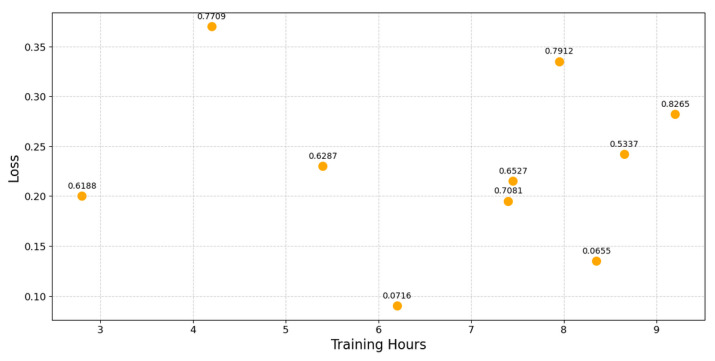
Training loss and accuracy over training time for SPN optimization.

Next, we investigated the relation between training time and LSTM model performance, using loss and accuracy as our metrics. Using data from the hyper-parameter optimization experiments for select SPN and PGN models, we were able to deduce that training time does not necessarily have a strong influence on minimizing loss and maximizing accuracy. Figure 8 represents the weak relationship between training time and loss of the optimization of SPN 191, where the dots are labelled with the accuracy rating.

**Figure 9:**
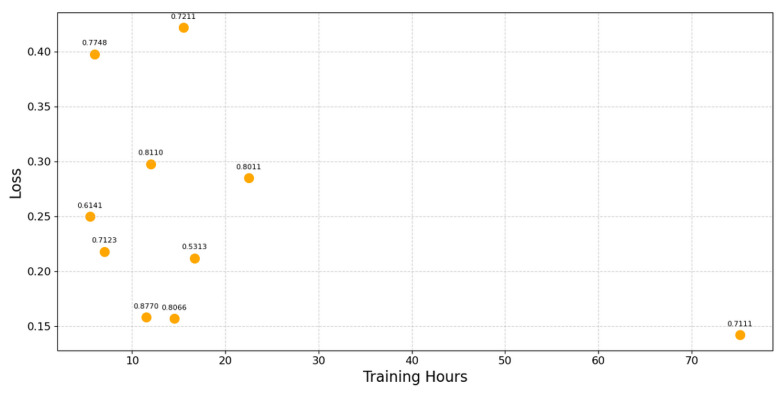
Training loss and accuracy over training time for PGN optimization.

Figure 9 displays the details for the optimization of PGN 0×F002. We would require more data to definitively make a conclusion regarding the correlations between network complexity and training time, as well as loss and accuracy.

However, we can deduce that LSTM models require a significant amount of experimenting and optimization to conduct the task it is trained to perform in the most effective and efficient way possible. In summary, we notice a trade-off between the effectiveness and efficiency of a model

### 3.4. Simulated J1939 Anomalies

The scenario behind our simulated attack is that one of the ECU nodes on the J1939 network has been compromised and is thus able to send malicious packets over the bus as a regular node in the vehicular network. This malicious node can send any data sequence from any ID to simulate anomalies. Thus, we can test both detection systems and set conditions to determine their effectiveness. By focusing on data sequence-related attacks, we imitate an adversary that modifies the Data field in J1939 packets to cause some malicious behaviour.

We used a modified version of the framework used by Taylor et al., described in Refs. [13,14], to generate two different types of malicious attacks: data replay and Data field modification. For a data replay attack, the payload of the Data field or sub-field that is being targeted is replaced by previously transmitted data from a different context than that of the preceding data sequences where the replayed data were captured. The Data field modification attack is the modification of the data payload of a particular ID; this technique is akin to the concept of a man-in-the-middle (MITM) attack.

### 3.5. Replay Attacks

An attacker wishing to execute a replay attack first requires the ability to capture traffic on the J1939 network. This allows them to gain an understanding of the context (cause and effect) of specific packets between ECUs. The attacker is then able to retransmit a previous packet to achieve a certain outcome. For example, a skilled attacker may monitor the traffic between the ECUs involved in the lane-keep-assist (LKA) sensors and the actual rotations of the steering wheel so that they can replay actions that are out of context and beyond the control of the driver. Several researchers have identified this type of attack as a serious threat to public safety, and it has been a priority of effort in the design and implementation of security countermeasures to mitigate the risk [8,27,33]. From the perspective of our attack scenario, we used traffic logs that were separate from the ones used to train our models to generate replay attacks. These data sequences were parameterized by model ID such that when the replay attack was activated, the next Data field in a sequence is replaced by the previous data from earlier in the traffic log. The data sequence is legitimate to its respective ID; however, it is incompatible with the preceding data.

### 3.6. Field Modification Attacks

Our second attack alters the individual sub-fields of the full data payload. These modifications are used to imitate attacks that set sub-fields to specific values to cause a desired effect. Like replay attacks, field modifications are parameterized by the model ID; they also consider characteristics such as the field to be affected and the duration of the modification. With regard to the attack framework by Taylor et al. [13,14], it is important to note that they did not explicitly understand the semantics of the subfields of the CAN bus packets; thus, they utilized the Markovitz and Wool field discovery algorithm [34] to configure this component of the attack scenario. For our purposes, as we focused on the SAE J1939 Standard, we were able to reconfigure the attack scenario with the actual data packet fields containing semantically valid information that would be out of context in a normal sequence.

We considered two types of field modifications to create anomalous data. First, the field could be set to a constant value by setting the potential values to the minimum/maximum of the data observed or to some other random values. Second, fields can be modified consecutively by replaying a sequence of previously transmitted values or by using random data sequences that fall outside of what has been observed in the data. The latter is like the replay attack; however, it is concentrated at the sub-field level, so it is included for completeness. It is important to note that even though our J1939 detector was trained at the sub-field level, we chose to keep the replay mode of the field modification attack in our experiments, as it was programmed for a specified duration of time, whereas the replay attacks were singular actions.

The simulated attacks were designed to realistically represent possible attacks against the CAN bus and J1939 to effectively test our detection systems. As we have seen in the vehicular security field, there are numerous attack vectors that can be used to change data values and cause a malicious effect. Thus, we found that our simulated attack was representative and allowed us to draw significant conclusions based on the results obtained for both detection systems.

### 3.7. Anomaly-Based Detection Architecture

**Figure 10:**
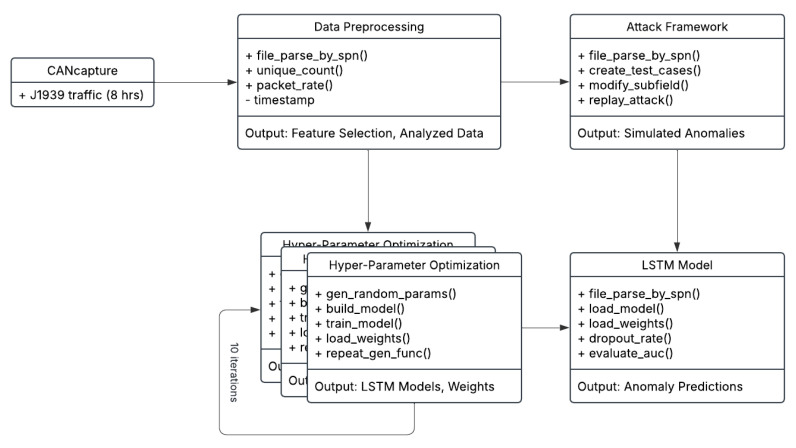
J1939 IDS architecture design model.

The design architecture of our J1939 proof of concept detection system is depicted below in Figure 10. After the traffic capture step, the data preprocessing phrase was conducted, which relied heavily on the use of various Python libraries such as NumPy 1.18.1, pandas 1.0.1, matplotlib 3.2.0 [35], as well as custom tools, to thoroughly analyze the dataset. Next, we conducted a process called feature engineering [24], which consists of feature selection and extraction, to ensure that the appropriate data subsets and features were chosen for building our LSTM models. The data analytics step also ensured that there was enough variability in the dataset to generate sufficient anomalies to evaluate the models.

Next, with our dataset analyzed and features selected, we iterated through the hyper-parameters optimization task to train and build the best LSTM models with the lowest log loss error calculated using the binary cross-entropy [24] Equation (12). The analyzed data also allows us to create test cases using the modified version of the attack framework designed by Taylor et al. [14] that we reconfigured for J1939 network systems.

(12) $$\log loss=-\frac{1}{n}\sum_{i=1}^{n}[y^{\left(i\right)}*\log\left({\hat{p}}^{\left(i\right)}\right)+\left(1-y^{\left(i\right)}\right)*\log\left(1-{\hat{p}}^{\left(i\right)})\right]$$

where *y* represents the true output, $\hat{p}$ represents the predicted output, and *n* is our batch sample in the training set.

After establishing our fine-tuned LSTM models and simulated attack data, we next cross referenced the models’ predictive function based on the actual anomalies generated so that we can determine the rate of true positives and false positives to yield a precision rate approaching, or equal to, one. Moreover, we extended our analysis to its decisive step by plotting the ROC curve and then computing the AUC score, thus allowing us to compare the various models accordingly.

In summary, our novel design for training LSTM models at the SPN sub-field level provides a unique approach to defending J1939 networks, which we then compared against a CAN bus detector in the subsequent section.

## 4. Experimental Results

### 4.1. Data Sequence Breakdown

All our experiment test cases were designed and implemented using real J1939 network traffic captured from a Class 8 truck. The eight driving hours logged were parsed into five separate text files that correspond to the five drives lasting approximately 1.5 h each. These files were divided into several subgroups: two files for training models, one file for the validation set of the training models, one file for the test set that contained both normal traffic and anomalies, and one file used to generate the replay attacks. Due to our constrained dataset, we used a different combination of files for each model ID for the preceding subsets.

The test set was divided into subsets of normal and anomalous cases. The data were broken down into three-second segments and then input into the attack framework to generate anomalies that simulated replay and data modification attacks. We generated three separate test sets for each experiment case. Ideally, we would have used the exact same anomalous data for each model to more substantially compare the results, but bandwidth and resource constraints prevented this. This limitation may have impacted the outcome and further reiterates the need to validate each model with actual attack data, which is left for future efforts and is outside the scope of this research.

### 4.2. Overall AUC Performance

The experimentation we conducted on our anomaly detection models allowed us to determine that the outcome was highly dependent on the type of attack that was implemented. Table 5 outlines the overall AUC values for each detection system, which displays the weighted average of all test cases; we also included an excerpt of the LSTM model results from the Subaru Impreza used by Taylor et al. in Ref. [13] for additional comparative analysis. Although we cannot make direct comparisons between the Class 8 truck we used and the Impreza used by Taylor et al., their results used a completely different network and dataset; Table 5 still provides us with a viable benchmark as to what constitutes a successful anomaly-based detection system for a vehicle network in such a closed test environment. Overall, we observe that our J1939 detector outperformed the physical layer CAN detector in most test cases based solely on AUC scores, except for ID 0×F005, which produced greater results than both its J1939 detector counterparts, SPN 526 and SPN 523. This may have been partly due to the reduced variability in the dataset that we identified in Section 3 or due to the behaviour of this node on the network.

### 4.3. Model Performance Metrics

In addition to comparing the AUC scores, we explored multiple techniques for the processing of the aggregate score types to calculate more complete results. We will carefully investigate the specific outcomes for our J1939 model based on the output score type, attack type, and duration of attack and compare them to the results of the CAN detection system. Table 6 displays our results according to the metrics we described to measure the effectiveness of our detection systems in Section 2; for each model, the decision threshold was set to maximize the *f_β_* score. The *f_β_* score for each model is in the 97–99% range, where β = 0.1, which gives a greater weight on precision than recall by an order of magnitude of 10. Overall, the SPN models are more effective than their PGN counterparts, except for the 0×F005 subgroup. This fact is illustrated by the high precision and f-score values, which signify that the SPN models function correctly by detecting anomalies with a relatively low false positive rate. In contrast, we observe that the PGN models yield higher rates of recall, indicating that these detectors identify a greater number of anomalies, albeit with a lower precision rate, yielding a higher false positive rate. It is important to note that even with a relatively high precision rate, such as 0.9975 with SPN 161, a significant number of false alarms would be generated that may require human engagement. For example, a fleet of 130 trucks would generate 1 trillion CAN messages in one year; with a precision of 0.9975, this translates to approximately 25 false alarms in 10,000 packets and a total of 2.5 billion false alarms in the truck fleet scenario above.

### 4.4. Results Breakdown by Attack Type

When we dig deeper into the attack representations of each test case, we see that the results differ for each detection system. Table 7 outlines the AUC values for each ID by attack type of both anomaly detectors. With regard to replay attacks, we observe that in most cases, the CAN detection models outperform the J1939 detector. There is one exception to this, the model for SPN 190, the parameter for actual engine speed, which greatly outperforms its corresponding CAN models by over 20% and yields one of the highest AUC values in this attack test case

When we look at the data modification attack types, we see that our model detects anomalies at a significantly higher rate than Taylor et al.’s CAN bus detector. The only exception to this is with the PGN model for 0×F005, the PG for gear ratios. This outcome is possible due to the discrete nature of the 0×F005 data subset that we discovered during our data analysis in Section 3. Looking at the specific PGs, for 0×F002, we see that SPN 191 and SPN 161 both outperformed the CAN detector counterpart by approximately 40% and achieved an AUC value greater than 0.95. These SPNs, which refer to transmission output/input shaft speed, respectively, are assessed to be valued highly enough to have their own dedicated security platform based on our research. Furthermore, the 0×F003 PG was generally outperformed by each J1939 system; however, SPN 3357 did not achieve significant results. This SPN pertains to the maximum available engine torque percentage and is bounded by a 0 to 100% data range. The other SPNs in this PGN performed slightly above average with greater than 0.75 AUC values. Moreover, the results for the PGN 0×F004 subgroup display a similar pattern. SPN 190, the engine speed in RPMs, clearly shows the highest AUC values of all attack methods. As well, SPN 512, the driver’s demand for engine torque percentage, outperforms the PGN model for most attack methods with above-average results. SPNs 513 and 2432 correspond to the actual engine torque and demand engine torque percentages, respectively, and also yield above-average results with greater than 0.75 AUC values. We noticed that any SPN sub-field that pertains to a percentage data range yields only above-average results; thus, it would be advantageous to dig deeper into the SAE J1939 protocol and analyze the relationships between SPNs in other PGs and test models against grouping multiple SPNs to test their effectiveness. In summary, upon investigating the specific data modification attacks, there is a definite distinction in the results of the CAN and J1939 detectors.

We conclude that the J1939 detector performed better for the PGN 0×F002 group, whereas the CAN detector was more effective for PGN 0×F005. For the other two IDs, PGNs 0×F003 and 0×F004, both detectors functioned effectively depending on the attack method. Furthermore, the results were dependent on the individual SPN sub-fields; thus, we determined that these two PGs would be most secure with a combination of both detection systems. Overall, both detectors are proven to be effective at identifying anomalies; however, the J1939 provides a finer grade of detection. Based on the design, our J1939 detector can identify the root cause of the abnormality since it focuses on the individual SPN sub-field. Because of this fact, it can take a more efficient countermeasure through an intrusion prevention system (IPS) or through digital forensics. Furthermore, these results suggest that our approach of utilizing SPN models in a specifically designed J1939 detection system can be more effective depending on the targeted SPN and more efficient for conducting IPS countermeasures and off-line forensics.

## 5. Discussion

### 5.1. Contributions

Up until recently, most security researchers have focused their efforts on securing the generic CAN bus. However, based on the open-source nature of the J1939 protocol, we assessed that the risk of a cyber-attack on a J1939 network is greater than for a consumer-grade, CAN-based network. The security and protection of all vehicle networks is critical for overall public safety. Moreover, there are also significant economic factors that may be negatively affected by a cyber-attack targeting commercial, heavy-duty vehicles that use the J1939 application layer protocol and that are the backbone of the supply chain system in North America [36]. Hence, the requirement is to defend the vehicle networks of heavy-duty trucks.

As a means of addressing this deficiency, we designed and implemented a J1939-specific anomaly-based detection system that utilizes LSTM neural network models at the SPN sub-field level. We compared its performance against a published CAN-based anomaly detector [13] that was configured to detect anomalies within the full 64-bit data payload. To ensure a controlled comparison, we utilized a simulated attack representation to test both our J1939 detector and his CAN detector on a J1939 network. In summary, based on our findings and contributions to the field, it would be prudent for security researchers to design a task-tailored system that is specifically geared toward application layer protocols, such as the SAE J1939, when applicable; however, a defense-in-depth approach is a highly recommended practice.

### 5.2. Future Work

We determined that with sufficient data logs, it is possible to train both detectors to identify anomalous data by predicting the next bit in the data sequence. Consequently, we tested each anomaly-based detection system by implementing the attack framework to determine the detection rates and overall effectiveness. Throughout our efforts, we identified numerous avenues for future work that were out of scope for this research but that would further the research of defending J1939 networks, such as the following:

- Reproduce experiments with larger datasets and utilize real attack data along with diverse types of attacks and compare results;
- Group related SPNs into one LSTM model to yield the potential for better results;
- Reconfigure IDS to allow deployment on a physical platform for real-time detection;
- Recreate experiments where LSTM models are trained based on the optimization of the hyper-parameters for every model ID.

### 5.3. Conclusions

In summary, our research determined that a J1939-based detection system is more effective on a case-by-case basis than its generic CAN detector. We implemented a systematic approach to test both detectors and evaluated their effectiveness by generating simulated anomalies using an attack representation designed by established security researchers. We conducted extensive traffic analysis to methodically select the appropriate features and parameters for our models. Throughout the research and investigation of previous works, we determined suitable model IDs to train, test, and validate to produce the optimal results for both our J1939 detector and a proven CAN detector [13]. There is no question that a J1939 network requires several layers of security and protective measures. Like with most complex systems, there is no one-size-fits-all, single solution to securing heavy-duty vehicular networks.

## Figures and Tables

**Table 1:** Breakdown of selected PGN packet counts.

PGN	# of Bits Changed	# of Packets	Unique Packets	Unique %
0×F002	34	2,080,170	1,224,093	58.85
0×F003	32	1,050,817	303,003	28.83
0×F004	49	1,050,819	684,908	65.18
0×F005	44	208,017	385	0.19

**Table 2:** Complete list of CAN IDs and J1939 IDs used for LSTM models.

CAN Detectors	J1939 Detectors
PGN 0×F002	SPN 191-Transmission Output Shaft Speed
	SPN 161-Transmission Input Shaft Speed
PGN 0×F003	SPN 91-Accelerator Pedal Position 1
	SPN 92-Engine Percent Load At Current Speed
	SPN 3357-Actual Maximum Available Engine-Percent Torque
	SPN 5398-Estimated Pumping-Percent Torque
PGN 0×F004	SPN 512-Driver’s Demand Engine-Percent Torque
	SPN 513-Actual Engine-Percent Torque
	SPN 190-Engine Speed
	SPN 2432-Engine Demand-Percent Torque
PGN 0×F005	SPN 526-Transmission Actual Gear Ratio
	SPN 523-Transmission Current Gear

**Table 3:** Hyper-parameter optimization results of SPN 191.

Batch Size	# of Hidden Layers	# of Hidden Units	Input Sequence	Dropout Rate	Training Loss	Validation Loss	Training Accuracy	Validation Accuracy
16	3	16	20	0.2	0.1943	0.1902	0.7081	0.7611
16	3	64	10	0.2	0.0981	0.1067	0.0716	0.0483
32	2	64	5	0.5	0.2150	0.1863	0.6527	0.8078
32	2	16	20	0.2	0.2012	0.2439	0.6188	0.6909
32	1	16	5	0.5	0.2325	0.3343	0.6287	0.5073
32	3	64	10	0.2	0.3367	0.3228	0.7912	0.8016
64	3	64	5	0.2	0.2837	0.2755	0.8265	0.8301
64	3	16	10	0.2	0.3705	0.4150	0.7709	0.7571
64	2	32	5	0.5	0.2420	0.2358	0.5337	0.5913
64	3	32	10	0.5	0.1343	0.1694	0.0655	0.1679

**Table 4:** Hyper-parameter optimization results of PGN 0×F002.

Batch Size	# of Hidden Layers	# of Hidden Units	Input Sequence	Dropout Rate	Training Loss	Validation Loss	Training Accuracy	Validation Accuracy
16	3	32	10	0.5	0.4216	0.4132	0.7211	0.7328
64	1	128	10	0.2	0.2946	0.2958	0.8110	0.8204
64	3	512	40	0.4	0.2869	0.3849	0.8011	0.7966
64	2	128	10	0.2	0.2123	0.2198	0.5313	0.5438
64	4	64	10	0.2	0.3975	0.4717	0.7748	0.6879
64	2	128	10	0.5	0.2173	0.2142	0.7123	0.8332
128	3	1024	60	0.2	0.1442	0.1580	0.7111	0.9582
128	2	32	10	0.5	0.2505	0.2719	0.6141	0.5839
256	1	512	20	0.2	0.1541	0.1642	0.8066	0.8798
256	1	62	10	0.5	0.1551	0.2343	0.8770	0.5917

**Table 5:** Overall AUC values for J1939 and CAN detection systems.

CAN Detector		J1939 Detector		CAN Detector from [13]	
ID	AUC	ID	AUC	ID	AUC
PGN 0×F002	0.6814	SPN 191	0.9459	002	0.9738
		SPN 161	0.9317	0D0	0.8023
PGN 0×F003	0.6164	SPN 91	0.8768	0D1	0.9307
		SPN 92	0.7820	0D4	0.9706
		SPN 3357	0.7733	140	0.9048
		SPN 5398	0.8822	141	0.8955
PGN 0×F004	0.7255	SPN 512	0.8068	360	0.8161
		SPN 513	0.7667	370	0.8070
		SPN 190	0.9927		
		SPN 2432	0.7837		
PGN 0×F005	0.9637	SPN 526	0.8624		
		SPN 523	0.8431		
Overall Average	0.7468	Overall Average	0.8539	Overall Average	0.8876

**Table 6:** Model performance for all IDs.

ID	Score Type	Threshold	Precision	Recall	*f_β_*
SPN 191	window	0.8967	0.9966	0.5973	0.9901
SPN 161	window	0.9330	0.9975	0.7986	0.9950
0×F002	max	0.9968	0.9942	0.5468	0.9862
SPN 91	max	0.9997	0.9883	0.7366	0.9850
SPN 92	window	0.8724	0.9982	0.3793	0.9824
SPN 3357	window	0.8438	0.9985	0.4540	0.9868
SPN 5398	window	0.9192	0.9977	0.6040	0.9913
0×F003	max	0.9618	0.9893	0.9433	0.9889
SPN 512	window	0.9827	1.0000	0.2967	0.9771
SPN 513	window	0.9038	0.9889	0.5327	0.9805
SPN 190	max	0.9995	1.0000	0.9687	0.9997
SPN 2432	window	0.9096	0.9849	0.6507	0.9799
0×F004	window	0.6898	0.9810	0.9570	0.9807
SPN 526	window	0.9985	0.9955	0.5933	0.9889
SPN 523	window	0.9913	0.9985	0.4293	0.9855
PGN 0×F005	window	0.1056	0.9964	0.9978	0.9934

**Table 7:** Test case AUC for replay and data modification attacks for both detectors.

CAN Detector			J1939 Detector		
ID	AUC Replay	AUC Data Mod.	ID	AUC Replay	AUC Data Mod.
PGN 0×F002	0.9760	0.6769	SPN 191	0.8688	0.9576
			SPN 161	0.8087	0.9527
PGN 0×F003	0.8368	0.6137	SPN 91	0.7873	0.8870
			SPN 92	0.6417	0.7914
			SPN 3357	0.6802	0.7397
			SPN 5398	0.7105	0.9175
PGN 0×F004	0.8009	0.7243	SPN 512	0.7368	0.8335
			SPN 513	0.6662	0.8049
			SPN 190	0.9625	0.9950
			SPN 2432	0.7111	0.8119
PGN 0×F005	0.9656	0.9637	SPN 526	0.7563	0.8969
			SPN 523	0.7005	0.8857
Overall Average	0.8948	0.7447	Overall Average	0.7526	0.8728

## Data Availability

The authors are open to sharing the data that lead to this study, with the concurrence of DRDC Valcartier.

## Abbreviations

The following abbreviations are used in this manuscript:

AD	Anomaly Detector
AUC	Area Under Curve
CAN	Controller Area Network
DoS	Denial-of-Service
DRDC	Defence Research and Development Centre
ECU	Electronic Control Unit
EV	Electric Vehicle
HLP	Higher Layer Protocol
IDS	Intrusion Detection System
IPS	Intrusion Prevention System
LSTM	Long Short-Term Memory
PG	Parameter Group
PGN	Parameter Group Number
RNN	Recurrent Neural Network
ROC	Receiving Operating Characteristic
SAEs	Society of Automotive Engineers
SPNs	Suspect Parameter Numbers
